# Assessing Knowledge and Use Practices of Plastic Food Packaging among Young Adults in South Africa: Concerns about Chemicals and Health

**DOI:** 10.3390/ijerph182010576

**Published:** 2021-10-09

**Authors:** Magdi Du Preez, Daleen Van der Merwe, Louise Wyma, Susanna Maria Ellis

**Affiliations:** 1 Africa Unit for Transdisciplinary Health Research, North-West University, Potchefstroom 2520, South Africa; magdidupreez@gmail.com (M.D.P.); louise.wyma@nwu.ac.za (L.W.); 2 Pure and Applied Analytics (PAA), North-West University, Potchefstroom 2351, South Africa; suria.ellis@nwu.ac.za

**Keywords:** plastic, food packaging, bisphenol A, consumer knowledge, health concern, information sources, utilization practices

## Abstract

Chemicals associated with health problems can migrate from packaging into food matrices. Therefore, consumers need to be aware of health concerns associated with incorrectly used plastic food packaging. However, little is known about consumers’ knowledge and their plastics usage practices. This study assessed this knowledge and practices among young South African adult consumers. Our online survey of 293 participants focused on their objective (actual) and subjective (self-perceived) knowledge about plastic food packaging care and safety, their utilization practices, and their sources of information about safe use of plastics. Participants’ utilization practices showed broad misuse. Their subjective knowledge about the correct use of plastic packaging was in most respects contradicted by their limited objective knowledge. We found that plastic identification codes on packaging largely failed in their informative purpose; instead, participants mainly consulted informal information sources about plastics. The knowledge gaps, unsafe plastic use practices, and information source deficiencies identified here can help to guide future improvements. We call for consumer education, across all demographics, about plastic utilization practices and associated health concerns about plastic chemicals. We also highlight the need for the government, food and plastics industries to join forces in ensuring that consumers are informed about safe plastic packaging usage.

## 1. Introduction

Food packaging is rapidly evolving [1]. Such packaging serves several purposes, including physically protecting food and beverages during production, transport and storing processes; safeguarding food from spoilage; and providing product information [1,2]. Though plastic is exceptionally versatile, it has a disturbing influence on environmental pollution [3] and poses significant economic and social risks [4]. Food packaging amounts to 42% of globally produced plastic material and has a very short lifetime of around six months [5]. In South Africa, packaging industries utilize 52% of local and imported plastic raw materials [4]. Global plastic waste from 1950–2015 is estimated at almost 7 billion tonnes of waste with a proposed 60% dumped in landfills [5]. While higher-income countries produce the most plastic waste, they also have efficient waste management infrastructure and systems [6]. However, sub-Saharan Africa countries inadequately dispose of 80–90% of plastic [7]. In South Africa 1,492,000 tonnes of plastic are locally produced [8], not including imported amounts. Plastics in this country are predominantly disposed of via landfills, with the bulk of recycling taking place by waste pickers from curbsides or landfills. However, formal recycling establishments and businesses are increasing [9].

Although much is written about plastic safety, dumping, and clinical uses, little research could be found on consumer utilization of plastic food packaging [10]. A recent review [11] confirmed that consumers are aware and informed of the need to avoid or reduce their use of plastic packaging materials. However, this is not always possible in practice, since the use of plastic for packaging has increased, and various food and beverage packaging applications and polymer modifications have been developed [12,13].

Concerns about plastic packaging safety have been reported, since plastic food and beverage packaging and containers—referred to in this study as plastic food packaging—interact with the food they contain [14,15]. Different types and amounts of chemicals with estrogenic properties migrate from packaging into the products it contains [16,17]. These chemicals include bisphenol A (BPA) and BPA alternatives, of which the latter have not yet been intensively studied [18,19]. According to Kasonga [20], producers globally fail to mention these chemicals in their chemical compound list. However, some polymers are indicated globally and voluntarily in South Africa by the plastic identification codes, although these codes do not indicate product safety [21]. Furthermore, available risk assessments relating to plastic packaging chemicals do not fully and sufficiently protect public health [17].

Consumers worldwide are most familiar with BPA [2], an endocrine-disrupting chemical (EDC) that alters homeostatic, hormonal and several biological systems [22,23]. Studies show that EDCs may be associated with disease and health problems [24] by interfering with hormonal actions, homeostasis, reproduction, and developmental processes [25,26]. Changes in the endocrine system can cause disease, undermine health, and even lead to death [15,27].

Despite the benefits of plastics, they can pose risks to human health and the environment [28] through existing production approaches and consumers’ utilization and disposal practices [18]. Although consumers express interest in BPA and its safety [28,29], studies reveal that they are unaware and uninformed about chemical substances in plastic [30] and the effect of these substances on health [28]. Consumers may not know, therefore, that plastic products harmful chemicals migrate from plastic products, and that persistent incorrect utilization practices may adversely affect their health. Plastic identification codes are provided on packaging to guide consumers on the safety of plastic products [31]. These identification codes developed by the American Society of the Plastics Industry (ASTM) are used internationally to identify the raw material composition of plastic products and do not indicate whether a product can be recycled. Therefore, internationally, these codes were replaced by the American Society for Testing and Materials (ASTM D7611–13) in 2013. However, many South African plastic manufacturers still use the original identification codes due to the high costs of changing plastic manufacturing moulds [4,32]. Still, how well these codes inform consumers remains uncertain.

Research in China showed that consumers rated their own knowledge about food packaging as below average [28]. Previous South African studies [33,34,35,36,37] have highlighted consumers’ lack of knowledge about nutrition and labelling, environmentally friendly packaging, and consumer protection, and these deficiencies could indicate a further possible lack of knowledge about plastic food packaging, resulting in its potential misuse. It has been shown that consumers’ utilization and disposal practices outweigh manufacturers’ impact on the environment [38]. The same may hold for the health-related dangers of consumers’ utilization of plastic food packaging. Furthermore, a general lack of sufficiently available information about plastics and BPA in emerging countries such as Cameroon and Nigeria [39] could contribute to sub-optimal consumer choices and practices relating to plastic food packaging. International research into consumers’ behaviour (e.g., perceptions, understanding, decisions) and plastic packaging is sparse and relates mainly to sustainability [10,11,35,40]. Furthermore, research on plastics and health concerns is abundant; however, only a few studies relate to consumers’ awareness of plastics and human health [28,41,42] and does not focus on plastic as food packaging. This gap is even more apparent in South Africa, where existing research pertains to plastic and sustainability [43] and no studies, thus far, focused on consumers and plastics or plastic packaging in the context of health.

The present study provides a baseline for understanding the nature and extent of the use and misuse of plastic packaging by young adult South African consumers, with a view to informing appropriate action should this be required. Our main aim was to investigate their knowledge about and approach to the safe use of plastic food packaging. Three research questions guided us: What are these consumers’ utilization practices relating to plastic food packaging? What objective and subjective knowledge do they have about the correct use of these products? What information sources do they consult to obtain information about the proper use of plastic packaging and about BPA and other harmful chemicals? These research questions are critical from a public health perspective. Given increasing health consciousness among consumers and their concern over the naturalness of food [44], it is in their best interest to use plastic packaging of food products safely, to avoid migration of chemical residues into what will be ingested, and to prevent their healthy eating efforts from being undermined. Healthy eating has long been seen as an effective way to stay healthy; it needs safeguarding from the harmful effects of plastic packaging misuse.

## 2. Chemicals in Plastic Food Packaging and Implications for Consumers

Consumers are exposed to various chemicals in plastic packaging materials used for food and other goods [15,23]. Most plastic products are reported to release or let different chemicals migrate into food in different amounts, depending on the storage time, temperatures and chemistry of the packaging and of the food items [16,17,45], with BPA posing a key concern. Plastics containing harmful chemicals are used to produce reusable beverage containers, frozen and prepared food containers, oven trays, microwavable cookware, and “boiling–in–the–bag” packs [16,46]. Roasting and microwaving food in such packaging accelerates the migration of chemicals [13]. In addition, epoxy resins used in coating canned food and drinks may also allow the migration of BPA and BPA alternatives [23,47,48]. Standard consumer utilization practices include refilling and reusing single-use plastic products, such as beverages and water bottles, that have not been designed for such standard reuse practices or assessed in terms of their effects [49].

We could not find specific evidence in the literature that consumers use packaging labels to inform their plastic use practices (e.g., microwaving). A study among Libanese consumers did not investigate packaging labels. However, it showed that participants’ high regard for packaging safety (i.e., stability and non-migrating into its content) did not culminate as a significant criterion during purchasing. They judged quality, safety and health based on packaging characteristics such as material and colour [50]. Australian research showed that consumers read and understood preparation and storage instructions of infant formula but neglected or only partially followed them [51]. Though not about package usage in particular, studies in South Africa indicated that consumers were likely to use the usage instructions on food labels during purchasing [52] and that the instructions affected most participants’ purchase decisions [29]. Another study confirmed label usage to preserve freshness and prepare the product [53]. Nevertheless, it is proposed that limited labelling availability, accuracy and credibility in South Africa may hamper consumers ability to make decisions on plastic recycling practices and purchase decisions [54]. We can deduce the same regarding safe packaging usage based on these labelling limitations.

The risks of long-term exposure and accumulation of harmful packaging chemicals remain uncertain and even unknown [15]. Studies show that even at low levels, EDCs influence bodily functions, thereby putting consumers’ future health at risk [27,47]. Nevertheless, only a few consumers have mentioned food packaging materials when asked about food contaminants [55]. Furthermore, consumers are still largely unaware of sources of food contamination other than bacterial [15]. They are also unaware that BPA-free products contain alternatives or substitutes—potentially as harmful as BPA [19,48]—and that “BPA–free” does not mean EDC-free [16,41]. Despite some consumers’ reported awareness of chemical substances, they remain uncertain about quantities of chemicals in products and the risks to human health [17,30]. A review has shown that although consumers are aware that they should avoid or reduce their use of plastic packaging, most research in this domain focuses on the environment [11]. Environmental risks need serious attention and investment, but Bornman et al. [38] emphasize the fact that EDCs are deserving of attention as a matter of public health and intervention.

Global advances regarding EDC regulations have been made to prevent the migration of harmful substances from food packaging materials into food products. These regulations, mostly in high-income countries, aim to provide safe plastic packaging materials to protect human health [15], especially relating to possible hormonal disruptive disorders from BPAs [48]. Canada was the first country to ban BPA use in baby bottles (2008), followed by a European Union ban in 2010 to protect this most vulnerable and exposed consumer group [23]. However, not only babies are susceptible to the risks of adverse health effects from high-level exposure to BPA and EDCs [23]. It is recommended that, instead of focusing only on the treatment of diseases caused by EDC exposure, African countries take similar action—including policy and practices—to stop exposure to EDCs in the first place [38].

In South Africa (an upper–middle-income country [56]), Erasmus [57] argues that most consumers are vulnerable due to knowledge and resource limitations. The first and only legislation regarding BPA in South Africa intervenes to protect infants and babies. South Africa became the first country in Africa with legislation banning BPA usage in baby bottles [58], including prohibiting the manufacturing, importation, exportation, and sale of infant bottles containing BPA. These regulations—R. 879 of 2011—are included in the Foodstuffs, Cosmetics and Disinfectants Act of 1972 [59]. However, this legislation only applies to baby bottles, not other plastic food packaging. South African consumers are also protected by the Consumer Protection Act of 2008, which aims to safeguard them against safety and health threats and improves information provision facilitating informed choices [60]. However, these consumers are not necessarily furnished to understand and apply the information provided [57].

Furthermore, it is difficult to clearly identify the presence of BPA in products [61]. Notwithstanding consumers’ familiarity with or understanding of plastic identification codes, consumers are exposed to BPA daily through basic commodities such as packaging, which are seldom given much thought [62,63]. They, therefore, need protection by legislation. In terms of environmental concerns and plastic packaging, South Africa promulgated new regulations in November 2020 concerning Extended Producer Responsibility (EPR) as part of the National Environmental Management: Waste Act of 2008 [64]. Complete monitoring and reporting for these regulations will start during the first quarter of 2022. They require mandatory EPR from packaging producers and importers, addressing role player product handling in the packaging chain from design to recycling [21]. Additionally, although the South African Bureau of Standards released a voluntary standard on the requirements for marking and identifying degradable packaging (SANS 1728), South Africa does not currently have mandatory regulations for environmental labelling [65] to assist consumers’ decision making.

Since current legislation does not include other plastic items containing BPA, and the presence of BPA and other potentially harmful chemicals are difficult to identify, South African consumers are left to fend for themselves by self-regulating legislation. Table 1 summarizes the leading plastic polymeric materials in South Africa and their characteristics in terms of producers, amounts, potentially harmful chemicals and health-related concerns.

## 3. Materials and Methods

This study was approved by the Health Research Ethics Committee of the North–West University, South Africa (reference number NWU–00338–16–A1).

### 3.1. Participants

This research was conducted across South Africa to reach consumers from different regions and demographics, allowing for as wide as possible variation in consumer perspectives. We used purposive sampling to select participants representing the specific population with the characteristics or attributes that best suited the study’s purpose [79], without the intention to generalize findings. The inclusion criteria specified that participants had to be South African citizens, aged between 18 and 35 years, who were not living with their parents, who used plastic packaging or containers, and who bought, prepared, and stored their own food. We expected that younger consumers (18–35 years) who had recently started taking care of themselves would be exposed to more complex food-related decisions (e.g., the type of packaged food to be purchased) than more experienced consumers. For these younger consumers, purchasing decisions may be high involvement decisions. First time purchasers may also be more likely to read food labels [80]. Furthermore, they still have to use plastic for an extended period. These young adults must transfer their knowledge and educate their children regarding plastic food and beverage container usage. We excluded participants working or studying in the food or packaging industries or chemical engineering, since they may have had advanced experience with the risks of misusing plastics. Recruitment was conducted between March and July 2017 through the Consulta Research Company to provide access to a broad range of consumers who met the inclusion criteria nationwide.

A total of 293 consumers participated, close to the sample size of 300 recommended by Tabachnick and Fidell [81] for factor analysis. We focused on a younger-aged group since they would recently have started taking care of themselves and making independent food decisions, and they may have found these responsibilities more challenging than older, more experienced consumers. Furthermore, determining their knowledge would assist, if necessary, in the task of educating consumers about plastic food packaging and transferring relevant knowledge to the next generation.

### 3.2. Data Collection

A questionnaire (Supplementary Materials) was compiled, based on the literature and on previous instruments measuring similar variables in other contexts, to address the research questions. It comprised seven sections, of which the first (Section A) related to the inclusion criteria.

Section B addressed the usage frequency of plastic food packaging by means of two Likert scales, followed by usage practices in which they engage. The first Likert scale had nine items for determining participants’ consumption of products from such packaging (1 = Never; 2 = Rarely; 3 = Monthly; 4 = Weekly; 5 = Daily) and the second scale had five items about their use of plastic identification codes (1 = Never, 2 = Rarely, 3 = Sometimes, 4 = Always). Following the Likert scales, we determined how frequently consumers used 11 different plastic products in different ways (e.g., for refrigeration, freezing, microwaving, in the dishwasher, sun exposure) adapted from Nomura et al. [42] and Havenga [82]. Section C assessed subjective knowledge (i.e., self-assessed knowledge or self-beliefs regarding their own knowledge [83,84]. We posed the statement to participants: “I know how to correctly use the following food and beverage packaging and containers”. They then had to indicate their level of agreement with this statement for 11 different kinds of plastic packaging (1 = Strongly disagree to 5 = Strongly agree). This scale was followed by the statement “Compared to an average person, I …” where they again had to indicate their level of agreement about five items regarding their plastic packaging buying behaviours and decisions. Section C was adapted from Malindi [85]. Section D assessed objective knowledge related to the care of different kinds of plastic food packaging (i.e., freezing, microwaving, dishwashing, reusing, exposure to extreme temperatures, and storing for prolonged periods) using Yes, No, and Don’t know options. We also assessed objective knowledge about the migration of chemicals from different packaging, illnesses/diseases linked to plastic packaging, plastic identification codes, and BPA (True, False, Don’t know) through questions adapted from Mongolia Baseline [86] and Havenga [82]. The percentage of participants’ correct responses was calculated according to set correct answers indicated in a predetermined literature-based memorandum. Section E addressed 13 information sources that participants may have consulted regarding plastic food packaging (Yes/No) and their trust in these sources (1 = Strongly disagree to 5 = Strongly agree). These questions were adapted from Havenga [82] and the European Commission Special Eurobarometer [87]. The final section (Section F) served to obtain demographic and general information. We conducted cognitive interviews with consumers not participating in the study before administering the questionnaire to establish whether potential participants of the study would understand the questions as intended.

Consulta Research Company invited participation via advertisements on their web page and social media crowdsourcing. The invitation was linked to an informed consent form. After providing informed consent, participants were linked to the online survey.

### 3.3. Statistical Analysis

For data analysis, we employed SPSS software version 22 (IBM Corporation, Chicago, IL, USA). Descriptive statistics, namely, frequencies, means, and standard deviations, were determined. Exploratory factor analyses (EFA) were conducted to establish construct validity. We used principal axis factoring with Oblimin rotation on Sections B to E. Cronbach alpha values were used to determine the internal reliability of all factors yielded by the EFAs, except for the dichotomous scales, where Kuder–Richardson 20 served this purpose. Mean factor scores were calculated for each factor, considering the means of all the items contributing to it. Two-sided independent *t*-tests, one-way analysis of variance (ANOVA), and Spearman’s rank-order correlations were also conducted for all constructs. Owing to our non-probability sampling method, we used effect sizes to indicate practical significance and tendencies. We only report Spearman’s rank-order correlations with large effect sizes (*r* = 0.5 large effect size) showing practically significant associations. For differences in means between demographic groups, only Cohen’s *d*-values indicating practical significance (*d* = 0.8 large) and medium effect sizes or tendencies (*d* = 0.5 medium) are reported [88].

## 4. Results and Discussion

### 4.1. Demographics and Self-Reported Health Profile

The participants’ demographic profile (*n* = 293; Table 2) indicates that more women participated in the study than men, though the latter were well represented. Over 90% were aged 26–35 years, 45.4% were white, and almost a third was African. Coloured and Indian participants were under-represented. Overall education levels were high, in that most were graduates with either a diploma, degree or additional postgraduate qualification, and almost all (above 90%) were employed full-time.

Most participants (80.7%) reported that their overall health was “good” and “excellent”, with less than one-fifth “fair” and “poor” (Table 2). The participants confirmed these results, almost 80% indicating that they suffered from “none” of the illnesses/diseases listed in the questionnaire, of which all have been linked or associated with BPA or EDCs in some way [74,89]. The participants, overall, enjoyed self-reported good health, probably related to their young age.

### 4.2. Results from Factor Analyses

The EFAs for Sections B, C, D and E explained an acceptable 42.0–71.2% of the variance (Table 3). Only EFAs relating to the frequency of food consumption from plastic packaging (35.9%) and objective knowledge about reuse practices (33.4%) had a low percentage variance explained. However, their factor structures theoretically made sense while they also had high KMO values of 0.76 and 0.77, respectively, and a Cronbach alpha and Kuder–Richardson 20 value of 0.78 and 0.80, respectively. In all instances, factors extracted yielded acceptable KMO values greater than 0.5 (KMO = 0.61–0.92) (Table 3) with confirmed construct validity. All factors showed acceptable internal reliabilities (Cronbach alpha/Kuder–Richardson 20 = 0.60–0.95) above 0.60 [90]. Furthermore, most factors’ inter-item correlations were within the recommended range of 0.15–0.55 [91]. Only “utilization practices of plastic identification codes” (5 items relating to these codes) and subjective knowledge about “utilization practices” (11 items relating to different practices) had inter-item correlations higher than 0.6, indicating that items within these factors had a higher level of similarity (Table 3).

### 4.3. Participants’ Utilization Practices of Plastic Food Packaging

Participants’ use of food plastic packaging showed high frequency, and most participants indicated using it “daily” (58.1%) or “more than once a week” (23.0%). Furthermore, the factor “frequency of food consumption from plastic packaging” had a mean factor score of 3.19 showing at least monthly use (1 = Never; 5 = Daily) (Table 4). These results confirm that plastic was popular as food packaging, with almost daily use in participants’ everyday lives and approximately monthly consumption of processed products and drinks from such packaging. However, the “utilization of plastic identification codes” (factor score = 1.58) (Never = 1; 4 = Always) occurred never or rarely. The discrepancy between the frequent use of plastic food packaging versus identification codes may have two explanations. First, regular users of these plastics may no longer deem these codes necessary. This phenomenon was seen among food-label users who use such labels only for first-time purchases [80]. Second, neglect of these codes may point to unfamiliarity or lack of interest in such information. Plastic identification codes mostly used in South Africa do not indicate product safety [21] or recyclability but instead provide the chemical composition [4,32]. However, neglecting to use these codes, whether unintentionally or intentionally, raises concerns since this is the only information source provided on the packaging that relates to plastics.

Table 4 summarizes the different utilization practices of different types of plastic packaging. More than half of the participants indicated that storing food in plastic packaging and containers inside refrigerators was common practice. Packaged food less frequently stored in the refrigerator were typically designed for other purposes, including freezing, immediate consumption (e.g., boxed meals) or storage at room temperatures (e.g., canned foods). Though common practice, storage of refrigerated food in plastic is controversial, as it has been reported that some new and used packaging materials may allow chemicals to migrate at a temperature as low as 4 °C [71,92]. Thus, there is a possible communication gap to consumers about the potentially harmful effects of refrigerated storage in plastic packaging not designed for such use.

Apart from products manufactured to be stored in the freezer (such as boxed meals = 28%; frozen foods = 79.5%), it was standard practice for a third to a half of the participants to freeze plastic beverage bottles (33.8%), plastic lunchboxes (33.4%) and plastic bags (49.1%) not generally intended to be frozen (Table 4). An additional 19.1% also froze products with cling wrap. However, these standard freezing practices for such packaged products (both intended and not intended to be frozen) may lead to migration of BPA and other EDCs [46,74]. Therefore, consumers may be misusing plastic through lack of information or by not reading information on plastic manufacturers’ packaging. Some practices (e.g., freezing lunchboxes and water bottles) may stem from poor habits and ignorance. Sanlier [93] showed that young adults’ perceptions of food safety risks were “low”, resulting in incorrect storing, preparing, thawing, and reheating practices during food preparation. Studies have shown that consumers’ attitudes towards use of plastic and littering need changing [28]. We add to those findings that poor utilization habits and ignorance about plastic packaging must also be addressed in line with safety recommendations.

Among our participants, as expected, we found it was common practice to microwave pre-packed microwave meals (41.7%). However, microwaving lunchboxes (43.7%), plastic and polystyrene takeaway containers (24.2%), and frozen foods (21.8%) in plastic packaging were also common, and more than 10% of them microwaved products wrapped with cling wrap and in plastic bags. The use of convenience-related plastic food packaging in the microwave, not intended for this purpose, may stem from consumers’ and plastic manufacturers’ ignorance, as well as misleading or contradictory labelling. For example, we noted packaging with pictures instructing users to pierce the protective film or cut a corner of the bag before microwaving, as well as small print instructions to remove the food from the packaging altogether before microwaving it. When available, the trustworthiness of plastic packaging labelling on recycling was shown as restrictive to South African consumers’ purchase and recycling behaviour [54]. Plastic labelling informing consumers about safe usage practices seems to be subject to similar limitations.

Infants and children are more vulnerable than adults to BPA and EDC exposure, because of the amount of BPA- and EDC-exposed food per kilogram of body weight [46]. The smaller the package, the higher is the surface-to-volume ratio of chemical migration [17]. Some plastic food packaging that may endanger health is specifically designed for children’s use (e.g., decorated lunchboxes) and should not be stored in the refrigerator, frozen, or heated. To maintain their different properties and quality, nearly all the packaging and containers listed in Table 4 should not be frozen, microwaved, washed in a dishwasher, or exposed to sunlight for long periods. In our study, without realizing it, many participants misused plastic storage bags, cling wrap, takeaway containers, and lunchboxes through one or more incorrect utilization practices, probably because they did not know about the potential health effects or through incorrectly learned behaviour. Misuse included exposing plastic to extreme temperatures, such as washing lunchboxes (32.8%) or plastic beverage bottles (8.9%) in dishwashers, exposing these items for prolonged periods to sunlight (8.2% and 12.6%, respectively), or microwaving or freezing them (Table 4). The reported migration of BPA and EDCs from plastics in extreme temperatures [71] makes exposing food in plastic packaging to such conditions potentially harmful to human health. Migration has been observed at 24 °C (room temperature) and between 40–100 °C (heating and cooking temperatures) [71,92]. Thus, the alarming level of participants’ misuse of lunchboxes and beverage bottles raises concern.

### 4.4. Subjective Knowledge about Plastic Food Packaging

Research among Chinese participants has shown low self-rated knowledge about plastic food packaging and its safety [33]. Similarly, our participants’ subjective knowledge about their ability to execute sound “plastic buying decisions” was neutral (factor score = 2.69 where 1 = Strongly disagree and 5 = Strongly agree). However, they had confidence in their knowledge of proper plastic packaging “utilization practices” (factor score = 3.63) (Table 4), thereby revealing higher confidence about their knowledge of plastic packaging utilization practices than about their ability to make informed buying decisions relating to these plastics. Participants’ limited subjective knowledge about “plastic buying decisions” may affect their purchasing of plastic food packaging.

However, participants’ confidence about appropriate use of different plastic packaging, did not, in most instances, translate into correct and safe utilization practices—hence, they probably overestimated their subjective knowledge. Although subjective knowledge points only to individuals’ own idea of how much they know [94], it is a strong driver of consumer behaviour [95].

The discrepancy between the two sets of subjective knowledge findings could be explained by the fact that, for high-frequency users of plastic packaging, confidence may come from their common day-to-day experience of using and storing plastic packaging. However, in purchasing plastic food packaging, they may have ignored criteria such as quality, safety, and identification codes in their decision-making. Buying the correct, safe plastic food packaging, however, is as important as using the bought product safely.

### 4.5. Participants’ Objective Knowledge about Plastic Food Packaging

Table 3 shows participants’ mean percentage of correct responses to questions posed. They lacked objective knowledge (<50% correct responses) about: “plastic identification codes—reuse and recycle” (12% correct), “illnesses/diseases and the use of plastic products” (17%), “freezing of various plastics” (39%), “plastic products most often used in the microwave” (42%), “BPA characteristics” (43%), and “plastic identification codes—specific utilization practices” (48%). We infer from these findings that most participants lacked objective knowledge about BPA and health concerns, practices related to microwaving and freezing, and—especially significant—plastic identification codes, which offer the only information available on plastic food packaging. However, consumers may not note these codes because they are also unaware of plastic packaging-related concerns, such as BPA and associated health issues. A recent review found that consumers had less knowledge about plastics and their recycling than about glass, paper and cardboard [11]. Our findings add to our understanding of knowledge deficiencies among consumers about the correct and safe use of plastic food packaging.

Our study’s correct responses also identified areas of participants’ knowledge and practice that require improvement to prevent potential adverse health consequences: the “release of chemicals from plastic products into foodstuffs” (53%), “the use of plastic products in the dishwasher” (65%), “utilization of plastic and products containing plastic after exposure to sunlight” (66%), and “reuse practices of various plastic food packaging” (67%). However, more than 70% of participants were knowledgeable about “plastic products and exposure to extreme temperatures” (72%), “freezing of cans and boxes” (81%), and “plastic products not often used in the microwave” (92%). These factors involve knowledge that may be more common in the broader consumer community. Previous research has shown that, despite consumers’ concerns about the environmental impacts of plastic packaging, these concerns did not necessarily spill over to their purchasing behaviour [40]. The same may be the case with our participants’ poor plastic utilization practices, despite having some objective knowledge about plastic packaging.

Overall, participants’ objective knowledge about plastic packaging largely echoed their utilization and subjective knowledge deficiencies. Their inadequate objective knowledge about 9 of the 12 knowledge categories, unsurprisingly, included microwaving and freezing. Participants were well-educated and reported a low incidence of diseases/illnesses linked to BPA or plastic-related chemicals; however, they did not have sturdy objective knowledge in most respects or subjective knowledge about plastics decisionmaking. These findings echoed participants’ own misuse of plastic food packaging.

Spearman’s rank-order correlations did not indicate any practically significant correlations between subjective and objective knowledge categories. However, *t*-tests showed that participants with high subjective knowledge about “plastic identification codes” also had practically significantly more (*d* = 0.73) objective knowledge regarding BPA characteristics (61% correct) compared to those with poor subjective knowledge about BPA (38% correct). Therefore, our findings showed that high subjective knowledge about plastic identification codes was associated with sound objective knowledge about BPA and its characteristics. Similar patterns of medium effect sizes were seen regarding high subjective knowledge about plastic identification codes being associated with solid objective knowledge regarding these codes. Specifically, differences between those with high and low subjective knowledge were seen in their objective knowledge regarding “identification codes—reuse and recycle” (23% vs. 9%, respectively; *d* = 0.50) and “identification codes—specific utilization practices” (63% vs. 44%, respectively; *d* = 0.46). These findings emphasize the need for focused consumer education efforts relating to plastic identification codes, to help develop both objective knowledge and subjective knowledge in consumers (i.e., correct knowledge accompanied by self-confidence) about identification codes, BPA, and proper use of plastic packaging. However, current legislation regarding BPA only covers infant bottles [59]. Therefore, our finding on participants knowledge limitations emphasizes the need for BPA (and its’ alternatives) labelling legislation protection for all consumers and plastic packaging. The Consumer Protection Act propose improved access to information [60], which is currently not evident regarding plastic food packaging.

### 4.6. Information Sources Consulted about Plastic Food Packaging and Chemicals

Our findings [according to the mean factor scores (yes/no)], showed that more participants (42%) on average obtained information about plastic food packaging from “informal information sources” from their social environment (e.g., friends, family, media, and internet) than from “formal information sources” (12%) in the education environment (e.g., scientists, health professionals, books, classes, and courses) (Table 3). These South African findings echo those from other emerging countries (i.e., Korea, Turkey and India) where it is easy to access informal information sources from the social environment, primarily via electronic information searches [96,97,98]. Although research has shown informal sources (i.e., Google, friends/relatives/colleagues and social media) of information about plastic consumption and health also to be popular among participants in Europe, they consider scholarly articles equally important [28].

Our South African participants agreed (factor score = 3.76) that they had more “trust in formal information sources” (1 = Strongly disagree; 5 = Strongly agree) and were more neutral in their agreement to “trust in informal sources” (factor score = 3.15) (Table 3). These findings echo Al Mamun et al. [44], who found that information from informal sources was not trusted or deemed essential enough to change consumer behaviour. This discrepancy between more frequent consultation of informal information sources and greater trust in the formal sources can possibly be attributed to more limited or restricted access to formal sources—including the cost implications and time commitment required. However, provision of reliable information to consumers will become possible only once data on chemicals related to plastic packaging is made more publicly available [15,18].

We found practically significant correlations of “informal information sources” with both “formal information sources” (*r* = 0.42) and “trust in informal information sources” (*r* = 0.51). The use of informal sources—which participants relied on more than formal sources—was associated with their trust in these sources. However, informal sources may not necessarily be trustworthy [99]. However, the correlation between the consultation of informal and formal sources is somewhat encouraging as it shows that participants with a high engagement in an informal information search may also be engaged in a formal information search process.

### 4.7. Demographic Differences for Participants’ Subjective and Objective Knowledge, Utilization Practices and Information Sources of Plastic Food Packaging

ANOVA and *t*-tests showed no practically significant differences between participants’ subjective and objective knowledge, utilization practices, and information sources from different demographic subgroups. However, *t*-tests did reveal medium effect sizes indicating tendencies of gender differences. A recent study in China found no gender differences in the purchase intent of plastic packaging [33]. In our study, however, we found gender differences, but only for objective knowledge. We identified female participants’ tendency to have more objective knowledge than males about “plastic products and exposure to extreme temperatures” (72% and 58%, respectively; *d* = 0.40) and “freezing of cans and boxes” (86% and 74%, respectively; *d* = 0.38). This difference may be due to learned behaviour, since females may have more experience than males of food purchase and preparation.

### 4.8. Limitations and Future Research

This study provided a baseline for assessing misuse practices and knowledge levels of young adults in South Africa about plastic food packaging and associated practices. The findings are not generalizable, but they have global interest since consumer usage practices relating to plastic packaging are important from a public health perspective. Furthermore, our study’s intentional focus on the younger demographic means that findings cannot be extrapolated to older consumers. Their young age and low-reported incidence of plastic-related illnesses/diseases, however, made them an ideal target population since they may be at risk for these diseases later in life if their misuse of plastics remains unaddressed. Additionally, as young consumers, they represent consumers of the future and a demographic taught by their elders and older role models at home. Therefore, their plastic handling and knowledge deficiencies may imply broader deficiencies than just in this population—especially since they were well educated overall. Future research should, thus, investigate plastic packaging-related behaviours among older consumers and those with lower education levels. Although we included employment status in our research, future research should also incorporate household income levels associated with health—their food decisions, such as nutrition label reading and knowledge [36].

## 5. Conclusions and Recommendations

Despite limitations and concerns related to the safe use of plastic products, they are widely used by consumers and the food packaging industry. The purpose of our study, therefore, was to assess consumers’ utilization of plastic food packaging, their knowledge about it, and the information sources they consult, in order to understand how such packaging is used and to inform interventions that may be necessary to guide safe and healthy practices in future.

Our study’s main contribution is to highlight severe deficiencies in our participants’ general dealings with plastic packaging. We identified poor utilization practices and the potential associated risks. We found that they were insufficiently informed (poor objective knowledge) about crucial health concerns related to unsafe use of plastic packaging and migration of BPA and other chemicals, and about the plastic identification codes. We discovered that these codes do not always serve an informative and protective role, as they were either ignored or misunderstood by our participants. An additional concern was that, although participants trusted formal sources of information, in reality, they neglected these in favour of less reliable informal ones.

These deficiencies relating to ubiquitous plastic food packaging call for urgent interventions to improve consumers’ utilization practices, knowledge, and ability to make safe and informed plastic packaging purchase and usage decisions. Health-promotion strategies in the area of consumer education need to include the meaning and value of plastic identification codes, the risks of migrating BPA and other chemicals, and the risks to health from the misuse of plastic packaging. Although we found gender differences across some objective knowledge categories, no sub-group in our young adult sample excelled in proper plastic usage and knowledge. It follows, therefore, that health promotion education on plastic packaging could be relevant across all demographics.

A further recommendation from our study is for health authorities to evaluate and guide the use of plastics as food packaging materials in a joint effort with the food industry. Our findings that plastic identification codes appear ineffective in guiding consumers suggest the need for role players at government level to revisit these codes—as they did with previous strategies to revise food labelling—for clarity and to ensure consumer protection. Manufacturers can also play their part by making accurate and unambiguous information available on packaging, with improved pictures and symbols that are clear and understandable to the broad range of consumers. Finally, policies relating to BPA and EDCs (currently only applicable to BPA in baby bottles) need to be reviewed for better protection of the wider consumer population. In emerging countries, such as South Africa, such policies are essential to safeguard all consumers, including those with low literacy levels or who have limited access to and understanding of the available information about plastic packaging. Our study provides a starting point for practical action to inform and educate the public about the dangers, as well as the safest possible use, of the food packaging that form part of everyone’s everyday life.

## Figures and Tables

**Table 1:** Summary of plastic polymetric materials in South Africa and potential health concerns.

Main Polymeric Materials ^1^	South African Producers	% Produced ^2^ [8]	Potentially Harmful Chemicals That May Be Present (Globally)	Health Issues Associated with BPA ^3^, BPA Alternatives and EDCs ^4^ (Globally)
Polyethylene terephthalate (PET)	Hosaf—polymerise PET from imported chemicals [66] Vinmar [67] Safripol [68]	12.7	Dibutyl phthalate (DBP)—EDC [69]. Di-(2ethylhexyl) phthalate (DEHP)—EDC [69] BPA and BPA alternatives—EDC [19,20] Antimony—Carcinogenic [70]	**Neurological and neuro-developmental effects and behavioural changes**—including attention deficit hyperactivity disorder (ADHD) [15,71,72]
High-Density Polyethylene (PE–HD/HDPE)	Sasol—high-purity ethylene gas [73] Safripol—raw material [66] Vinmar [67] DOW [74]	15.2	Polyethylene’s simple plastic polymer chemical structure could cause unhealthy hor-monal effects in humans [75].	**Hormonal effects in females**—Polycystic Ovarian Syndrome (PCOS), miscarriages, endome-trial disorders, infertility, obesity, female reproductive hormones, sexual behaviours [71,72]
Polyvinyl chloride (PVC)	Vinmar [67] Sasol [73]	10.5	Chlorine—carsinogenic [76] BPA and BPA alternatives—EDCs Phthalates—EDCs and carcinogenic [75].	**Hormonal effects in males**—affects the male reproductive tract, prostate cancer, testicular cancer, and a decrease in semen quality and sperm count in males [71,72,77]
Low-Density Polyethylene (PE–LD/LDPE)	Sasol—raw material using high purity ethylene [66] Vinmar [67] DOW [74]	22.8	Polyethylene’s simple plastic polymer chemical structure could cause unhealthy hormonal effects in humans. [75].	**Immune effects**—may negatively affect the immune system in general, immune cell population, proteins and genes expression, immune response [15,72,75]
Polypropylene (PP)	Sasol—propylene gas [73] DOW—propylene gas [74] Vinmar [67] Safripol [68]	21.1	Possible EDCs [75]	**Cardiovascular effects**—higher risks for cardiovascular diseases and atherosclerosis in adults [71], cardiac structures and functions, blood pressure [72]
Polystyrene (PS)	Raw materials are not manufactured locally, but this large industry produces various products and contributes to job creation and wealth [66]. Many companies manufacture products from imported polymers.	3.4	Styrene—Toxic to the brain and nervous system [75] Phthalates, Alkylphenols, Visphenol A, Di(2–ethylhexyl)adipate—EDCs [78]	**Metabolic effects**—Increased prevalence of obesity and diabetes [77] insulin signaling, glucose uptake, influences metabolic hormones and enzyme functions [72]
Other/Multi-layered Plastics Polycarbonate (PC) ^5^	Produced locally by many manufacturing companies.	14 (3.2% Polyurethane; 10.8% “others”)	Vast possibilities due to undefined materials.	**Genotoxicity**—Effects on chromosomal abnormalities and alignment as well as gene expression alterations, proteins and genes expression [70,72,77] **Carcinogenicity**—increase in breast, prostate, and testicular cancer [70,78] **Organ Failure**—health problems related to lungs and the liver [72,75]

^1^ Some of them may be BPA free. ^2^ % of the total 1,492,000 tonnes produced [8]. ^3^ BPA = Bisphenol A. ^4^ EDC = Endocrine-disrupting chemical. ^5^ Acrylonitrile butadiene styrene (ABS) or all other resins and multi-materials not otherwise defined.

**Table 2:** Frequencies and distributions of participants’ self-reported demographics and health.

Demographic Characteristics		Frequency (*n*)	%
Gender	Female	175	59.7
	Male	118	40.3
Age	18–25 years	18	6.1
	26–35 years	275	93.9
Ethnicity	African	94	32.1
	Asian	3	1.0
	Coloured	32	10.9
	Indian (descending from India)	23	7.8
	White	133	45.4
	Other	1	0.3
	Prefer not to say	7	2.4
Education	No education/some secondary schooling	1	0.3
	Complete secondary schooling (passed grade 12)	47	16.0
	Undergraduate (currently busy with post-school studies)	45	15.4
	Graduate (degree or diploma)	111	37.9
	Postgraduate degree	84	28.7
	Unclassified	5	1.7
Employment status	Employed (full-time)	269	91.8
	Employed (part-time)	12	4.1
	Unemployed	7	2.4
	Not applicable	5	1.7
Self-reported health	Poor	8	2.7
	Fair	47	16.0
	Good	172	58.7
	Excellent	66	22.0
Known illnesses or diseases of participants	Cardiovascular diseases	4	1.4
	Metabolic disorders (including diabetes type 2)	3	1.0
	Attention deficit hyperactivity disorder (ADHD)	5	1.7
	Urogenital abnormalities (urinary track defects)	1	0.3
	Infertility	6	2.0
	Don’t know	23	7.2
	None	233	79.5

**Table 3:** Summary of the different factors extracted with exploratory factor analyses.

Questionnaire Section	Factors Extracted from Factor Analysis	% Variance	KMO ^1^	Inter-Item Correlation	Cronbach Alpha/KR 20 ^2^	Mean Factor Score	SD ^3^
Section B: Utilization practices ^B^	Utilization of plastic identification codes	83.44	0.83	0.63	0.90	1.58	0.72
	Frequency of food consumption from plastic food packaging	35.87	0.76	0.31	0.78	3.19	0.62
Section C: Subjective knowledge ^C^	Utilization practices	66.24	0.92	0.63	0.95	3.63	0.82
	Plastic buying decisions	61.43	0.77	0.52	0.84	2.69	0.82
Section D: Objective knowledge ^D^	Freezing of various plastics	53.64	0.69	0.23	0.60	39%	0.30
	Freezing of cans and boxes			0.38	0.71	81%	0.28
	Plastic products not often used in the microwave	48.79	0.72	0.46	0.77	92%	0.21
	Plastic products most often used in the microwave			0.25	0.70	42%	0.26
	Use of plastic products in the dishwasher	44.79	0.78	0.35	0.81	65%	0.28
	Reuse practices of various plastic food packaging	33.38	0.77	0.31	0.80	67%	0.27
	Plastic products and exposure to extreme temperatures	42.03	0.85	0.36	0.86	72%	0.27
	Utilization of plastic and products containing plastic after exposure to sunlight	54.01	0.88	0.49	0.91	66%	0.34
	Release of chemicals from plastic products into foodstuffs	54.20	0.90	0.50	0.92	53%	0.36
	Illnesses/diseases and the use of plastic products	60.28	0.89	0.55	0.91	17%	0.28
	Plastic identification codes—reuse and recycle	65.47	0.61	0.42	0.59	12%	0.23
	Plastic identification codes—specific utilization practices			0.31	0.64	48%	0.42
	Bisphenol A (BPA) characteristics	46.55	0.84	0.38	0.85	43%	0.31
Section E: Information sources	Formal information sources	44.55	0.72	0.24	0.69	12%	0.19
	Informal information sources			0.41	0.73	42%	0.36
	Trust in formal information sources	57.63	0.83	0.43	0.81	3.76	0.58
	Trust in informal information sources			0.44	0.80	3.15	0.64

^1^ KMO = Kaiser–Meyer–Olkin test for sampling adequacy. ^2^ KR 20 = Kuder–Richardson 20 for knowledge and information source questions. ^3^ Standard deviation. ^B^ Utilization practice mean: Plastic identification codes: 1: Never; 2: Rarely; 3: Sometimes; 4: Always. Frequency of consumption mean: 1: Never; 2: Rarely; 3: Monthly; 4: Weekly; 5: Daily. **^C^** Subjective knowledge: 1 = Strongly disagree; 2 = Disagree; 3 = Neither agree nor disagree; 4 = Agree; 5 = Strongly agree. **^D^** Objective knowledge mean factor score presented as percentage correct response. **^E^** Information sources mean factor score presented as percentage “yes” responses to using a specific source.

**Table 4:** Frequencies of participants engaging in different utilization practices of plastic food packaging (*n* = 293).

Utilization Practices												
	None		Refrigerator		Freeze		Microwave		Dishwasher		Prolonged Exposure to Sunlight	
	*n*	%	*n*	%	*n*	%	*n*	%	*n*	%	*n*	%
Food cans (e.g., vegetables, fish, meat, sauces)	165	56.3	99	33.8	19	6.5	16	5.5	2	0.7	11	3.8
Boxed meals/meal in a box (e.g., microwave chips)	126	43.0	94	32.1	82	28.0	100	34.1	4	1.4	3	1.0
Cling wrap	112	38.2	163	55.6	56	19.1	43	14.7	0	0	4	1.4
Pre-packed microwave meals/convenience food	101	34.5	109	37.2	123	42.0	122	41.6	2	0.7	2	0.7
Plastic bags (e.g., sandwich)	92	31.4	168	57.3	144	49.1	34	11.6	2	0.7	6	2.0
Polystyrene or plastic take-away packaging	89	30.4	182	62.1	19	6.5	71	24.2	3	1.0	8	2.7
Plastic lunchboxes	40	13.7	211	72.0	98	33.4	128	43.7	96	32.8	24	8.2
Beverage cans (e.g., soft drinks)	36	12.3	249	85.0	20	6.8	3	1.0	1	0.3	16	5.5
Frozen foods in plastic packaging (e.g., vegetables)	17	5.8	103	35.2	233	79.5	64	21.8	1	0.3	3	1.0
Plastic beverage bottles (e.g., water, soft drinks)	11	3.8	273	93.2	99	33.8	23	7.8	26	8.9	37	12.6

Table sorted from highest to lowest, according to the “none” option.

## Data Availability

Data available on request due to restrictions, e.g., privacy or ethical.

## Supplementary Materials

The following are available online at https://www.mdpi.com/article/10.3390/ijerph182010576/s1, File S1: Questionnaire.
