# Supplementary material for: Assessing Knowledge and Use Practices of Plastic Food Packaging among Young Adults in South Africa: Concerns about Chemicals and Health
Source: Int J Environ Res Public Health. 2021 Oct 9;18(20):10576. doi: 10.3390/ijerph182010576 (PMC8535462; doi:10.3390/ijerph182010576)
Supplement: Supplementary file 1 [file ijerph-18-10576-s001.zip › ijerph-1375485-supplementary.pdf]

Supplementary File S1

Questionnaire

A: INCLUSION CRITERIA

1. **Do you make use of plastic food and beverage packaging and containers?**
   a) Yes
   b) No

2. **Do you or your spouse work as one of the following**

	Yes	No
a. Consumer Scientist		
b. Chemical engineer		
c. Nutritionist		
d. Packaging industry		

3. **Are you between 18 – 35 years old?**
   a) Yes
   b) No

4. **Year of birth____________________**

5. **Do you live with your parents?**
   a) Yes
   b) No

B: USE PRACTICES

6. How often do you make use of plastic food and beverage packaging and containers?

a) Daily
b) Once a week
c) More than once a week
d) Monthly
e) Occasionally
f) Never

7. Please mark the most relevant option for the following questions:

		Never	Rarely	Sometimes	Always
7.1.	How often do you make use of the following codes on plastic food and beverage packaging and containers?<br>(e.g 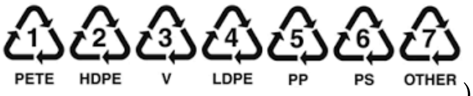)				
7.2	How often do these codes affect your decision to buy a product?				
7.3	How often do you use identification codes before you purchase a plastic product for the first time?				
7.4	How often do you read identification codes before reusing a plastic packaging or container?				
7.5	How often do identification codes affect the way you use the plastic packaging or container?				

8. How often do you eat foods, and consume drinks that are from the following containers?

		Never	Rarely	Monthly	Weekly	Daily
8.1	Plastic beverage bottles (e.g. water, soft drinks, ice tea, juice)					
8.2	Beverage cans (e.g. soft drinks, ice tea, juice, beer)					
8.3	Food cans: (e.g. vegetables, fish, meat, sauces)					
8.4	Prepacked microwave meals / convenience food (e.g. microwave spinach, pasta)					
8.5	Prepacked mixes: (e.g. muffins, cake, cupcakes, bread, pancakes)					
8.6	Boxed meals / meal in a box: (e.g. microwave chips, curry and rice)					
8.7	Plastic lunch boxes					
8.8	Frozen foods in plastic packaging: (e.g. vegetables, chips)					
8.9	Polystyrene or plastic take away packaging					

9. For each product type, select all the ways that you have used the product by ticking the relevant blocks

		Store in the fridge	Freeze	Micro-wave	Wash in dishwasher	Expose to sunlight for a long time	None
9.1	Plastic beverage bottles (e.g. water, soft drinks, ice tea, juice)						
9.2	Beverage cans (e.g. soft drinks, ice tea, juice, beer)						
9.3	Prepacked microwave meals / convenience food						

	(e.g. microwave spinach, pasta)						
9.4	Boxed meals / meal in a box: (e.g. microwave chips, curry and rice)						
9.5	Plastic lunch boxes						
9.6	Polystyrene or plastic take away packaging						
9.7	Food cans: (e.g. vegetables, fish, meat, sauces)						
9.8	Frozen foods in plastic packaging: (e.g. vegetables, chips)						
9.9	Cling wrap (e.g. Glad wrap)						
9.10	Plastic bags (e.g. Ziplock, Glad)						
9.11	Plastic shopping bags (e.g. Pick 'n Pay, Shoprite, Spar, Woolworths)						

C: SUBJECTIVE KNOWLEDGE

10. To which extent do you agree to the following for each type of packaging provided:
WHEN I LOOK AT THE PACKAGING, I KNOW HOW TO CORRECTLY USE THE FOLLOWING FOOD AND BEVERAGE PACKAGING AND CONTAINERS:

		1. Strongly disagree	2. Disagree	3. Neither agree nor disagree	4. Agree	5. Strongly agree
10.1	Plastic beverage bottles (e.g. water, soft drinks, ice tea, juice)					
10.2	Beverage cans (e.g. soft drinks, Ice tea, juice, beer)					
10.3	Prepacked microwave meals / convenience food (e.g. microwave spinach, pasta)					
10.4	Boxed meals/ meal in a box: (e.g. microwave chips, curry and rice)					
10.5	Plastic lunch boxes					
10.6	Polystyrene or plastic take away packaging					
10.7	Food cans: (e.g. vegetables, fish, meat, sauces)					
10.8	Frozen foods in plastic packaging: (e.g. vegetables, chips)					
10.9	Cling wrap (e.g. Glad wrap)					
10.10	Plastic bags (e.g. Ziplock bags, Glad)					
10.11	Plastic shopping bags (e.g. Pick 'n Pay, Shoprite, Spar, Woolworths)					

11. Compared to an average person, I …

		1. Strongly disagree	2. Disagree	3. Neither agree nor disagree	4. Agree	5. Strongly agree
11.1	…do not compromise on the quality of plastic that I buy and use					
11.2	…give myself enough time to buy plastic products, as its quality is important to my health					
11.3	…don't buy plastic food and beverage packaging and containers from unknown brands or suppliers					
11.4	…look for the plastic identification codes before I buy plastic food and beverage packaging and containers					
11.5	…evaluate different types of plastic food and beverage packaging and containers critically and regularly					

12. How would you rate your overall knowledge about food and beverage packaging and containers?

a) Poor
b) Fair
c) Good
d) Excellent

D: OBJECTIVE KNOWLEDGE

13. According to your knowledge, may the following food and beverage packaging and containers be frozen:

		Yes	No	Don't know
13.1	Plastic beverage bottles			
13.2	Beverage cans			
13.3	Food cans			
13.4	Plastic take away boxes			
13.5	Polystyrene take away boxes			
13.6	Lunch boxes			
13.7	Plastic bags (e.g. Ziplock, Glad) cook in bags, sandwich bags, freeze in bags			
13.8	Plastic shopping bags			
13.9	Cling wrap (e.g. Glad wrap)			

14. According to your knowledge, may the following food and beverage packaging and containers be heated in the microwave?

		Yes	No	Don't know
14.1	Plastic beverage bottles			
14.2	Beverage cans			
14.3	Food cans			
14.4	Plastic take away boxes			
14.5	Polystyrene take away boxes			
14.6	Lunch boxes			
14.7	Plastic bags (Ziplock, Glad) cook in bags, sandwich bags, freeze in bags			
14.8	Plastic shopping bags			
14.9	Cling wrap/ glad wrap			
14.10	Microwave meals			
14.11	Boxed meals			

15. According to your knowledge, may the following food and beverage packaging and containers be washed in a dishwasher:

		Yes	No	Don't know
15.1	Plastic beverage bottles			
15.2	Beverage cans			
15.3	Food cans			
15.4	Plastic take away boxes			
15.5	Polystyrene take away boxes			
15.6	Lunch boxes			

15.7	Microwave meal containers			
15.8	Boxed meal containers			

16. According to your knowledge, may the following food and beverage packaging and containers be used more than once:

		Yes	No	Don't know
16.1	Plastic beverage bottles			
16.2	Beverage cans			
16.3	Food cans			
16.4	Plastic take away boxes			
16.5	Polystyrene take away boxes			
16.6	Lunch boxes			
16.7	Plastic bags (e.g. Ziplock, Glad)			
16.9	Cling wrap/ glad wrap			
16.10	Microwave meal containers			
16.11	Boxed meal containers			

17. According to your knowledge, may the following food and beverage packaging and containers be exposed to different extreme temperatures (e.g. to cook food in and to freeze leftovers in)

		Yes	No	Don't know
17.1	Plastic beverage bottles			
17.2	Beverage cans			
17.3	Food cans			
17.4	Plastic take away boxes			
17.5	Polystyrene take away boxes			
17.6	Lunch boxes			
17.7	Plastic bags (Ziplock, Glad, sandwich bags)			
17.8	Plastic shopping bags			
17.9	Cling wrap/ glad wrap			
17.10	Microwave meal containers			
17.11	Boxed meal containers			

18. According to your knowledge, may the following food and beverage packaging and containers be used after it was left in the car for a long time:

		Yes	No	Don't know
18.1	Plastic beverage bottles			
18.2	Beverage cans			
18.3	Food cans			
18.4	Plastic take away boxes			
18.5	Polystyrene take away boxes			
18.6	Lunch boxes			
18.7	Plastic bags (Ziplock, Glad)			
18.8	Plastic shopping bags			
18.9	Cling wrap/ glad wrap			
18.10	Microwave meal containers			
18.11	Boxed meal containers			

19. According to your knowledge, can chemicals move from the following food and beverage packaging and containers into food and beverages:

		True	False	Don't know
19.1	Plastic beverage bottles			
19.2	Beverage cans			
19.3	Food cans			
19.4	Plastic take away boxes			
19.5	Polystyrene take away boxes			
19.6	Lunch boxes			
19.7	Plastic bags (e.g. Ziplock, Glad)			
19.8	Plastic shopping bags			
19.9	Cling wrap (e.g. Glad wrap)			
19.10	Microwave meal containers			
19.11	Boxed meal containers			
19.12	All plastic packaging and containers			

20. According to your knowledge, the following illnesses/ diseases may be linked to the use of plastic food and beverage packaging and containers:

		True	False	Don't know
20.1	Cardiovascular diseases,			
20.2	Liver enzyme abnormalities,			
20.3	Metabolic disorders (including diabetes type 2),			
20.4	Attention deficit hyperactivity disorder, (ADHD)			
20.5	Urogenital abnormalities (urinary track defects)			
20.6	Infertility			
20.7	Prostate cancer			
20.8	Breast cancer			

21. Please state if the following statements regarding plastic food and beverage packaging and containers are true or false:

		True	False	Don't know
21.1	Plastic beverage bottles may be used for warm beverages			
21.2	Cling wrap (e.g. Glad wrap) may be used in the microwave, if you poke holes into it.			
21.3	Food or beverage cans may be used to bake bread or pudding in			
21.4	Some food and beverage cans contain plastic			
21.5	Food may be heated in the can			

22. The following questions relate to the codes found on plastic packaging and containers:

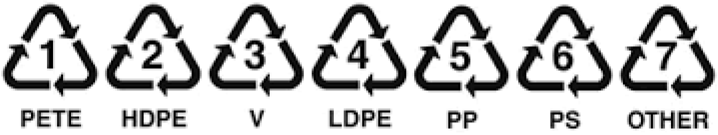

		True	False	Don't know
22.1	These codes show how many times a product may be used			
22.2	The codes show if a product is made out of recycled plastic			
22.3	The codes show that the product may be recycled			
22.4	The codes show what type of plastic is used in the product			
22.5	The codes show if a product may be heated			
22.6	The codes show if a product may be frozen			

23. The following questions relate to Bisphenol A (BPA)

		True	False	Don't know
23.1	BPA is a chemical substance			
23.2	BPA is a hormone			
23.3	BPA is a type of plastic			
23.4	BPA is found in plastic			
23.5	BPA is used in the production of certain types of plastics			
23.6	BPA is found in food and beverage cans			
23.7	BPA hardens plastic			
23.8	BPA makes plastic clear (see through)			
23.9	BPA is harmful to human health			

E: INFORMATION SOURCES

24. **Select all the sources that have provided you with information regarding the use practices of plastic food and beverage packaging and containers, and BPA**

		Yes	No
24.1	Scientists or academic researchers at universities		
24.2	Store assistants, supermarkets, retailers		
24.3	Health professions (e.g. doctors, nurses, dentists, chemists)		
24.4	Nutritionist / dietician		
24.5	Friends		
24.6	Family		
24.7	Classes / Course		
24.8	Media (e.g. Newspapers, Magazines, TV, Radio)		
24.9	Books		
24.10	Internet sources		
24.11	Government and legislation		
24.12	No one		
24.13	Other (pleases specify) ________________		

25. **I trust the information, regarding plastic and BPA, provided by these sources:**

		1. Strongly disagree	2. Disagree	3. Neither agree nor disagree	4. Agree	5. Strongly agree
25.1	Scientists or academic researchers at universities					
25.2	Store assistants, supermarkets, retailers					
25.3	Health professions (e.g. doctors, nurses, dentists, chemists)					
25.4	Nutritionist / dietician					
25.5	Friends					
25.6	Family					
25.7	Classes / Course					
25.8	Media (e.g. Newspapers, Magazines, TV, Radio)					
25.9	Books					
25.10	Internet sources					
25.11	Government and legislation					
25.12	No one					
25.13	Other (please specify) ________________					

F: BIOGRAPHICAL AND GENERAL INFORMATION:

Please select the most appropriate option for each question:

26. Please specify your highest level of education:
- a) No education
- b) Some primary schooling
- c) Complete primary schooling (passed grade 7/standard 5)
- d) Some secondary schooling
- e) Complete secondary schooling (passed grade 12/standard 10)
- f) Undergraduate (currently busy with after school graduate studies)
- g) Graduate (Degree or Diploma)
- h) Honours Graduate
- i) Masters graduate
- j) Doctors graduate
- k) Unclassified

27. What is your employment status?
- a) Employed (paid full time)
- b) Employed (paid part time)
- c) Pensioner/ Retired
- d) Self- employed (full time)
- e) Unemployed
- f) Not applicable

28. Please specify your ethnicity:
- a) African
- b) Asian
- c) Coloured
- d) Indian
- e) White
- f) Other
- g) Prefer not to say

29. Please specify your gender:
- a) Female
- **b)** Male

30. Please select the province in which your permanent residence is located:

a) Eastern Cape
b) Free State
c) Gauteng
d) Kwazulu Natal
e) Limpopo
f) Mpumalanga
g) North West
h) Northern Cape
i) Western Cape
j) Prefer not to say

31. For whom do you prepare food?

a) For the entire household
b) For yourself and children
c) Only for yourself
d) None of the above

32. How would you rate your overall health?

a) Poor
b) Fair
c) Good
d) Excellent

33. Do you suffer from any of the following illnesses? Select all the options that apply to you:

a) Cardiovascular diseases,
b) Liver enzyme abnormalities,
c) Metabolic disorders (including diabetes type 2),
d) Attention deficit hyperactivity disorder, (ADHD)
e) Urogenital abnormalities (urinary track defects)
f) Infertility
g) Prostate cancer
h) Breast cancer
i) None of the above
j) Don’t know
k) Other. Please specify________________

You have reached the end of the questionnaire. Thank you for your participation.
